# Randomised controlled trial of lifestyle interventions for abdominal obesity in primary health care

**DOI:** 10.1017/S1463423624000069

**Published:** 2024-04-19

**Authors:** Pedro Carrera-Bastos, Björn Rydhög, Maelán Fontes-Villalba, Daniel Arvidsson, Yvonne Granfeldt, Kristina Sundquist, Tommy Jönsson

**Affiliations:** 1 Center for Primary Health Care Research, Department of Clinical Sciences, Lund University, Malmö, Sweden; 2 Center for Health and Performance, Department of Food and Nutrition, University of Gothenburg, Gothenburg, Sweden; 3 Sport Science, Faculty of Education, University of Gothenburg, Gothenburg, Sweden; 4 Department of Food Technology, Engineering and Nutrition, Lund University, Lund, Sweden

**Keywords:** obesity, cardiovascular disease, disease prevention, cereal grains, physical exercise, waist circumference

## Abstract

**Aim:**

Assess effects on waist circumference from diet with or without cereal grains and with or without long-term physical exercise.

**Background:**

Elevated waist circumference is an indicator of increased abdominal fat storage and is accordingly associated with increased cardiovascular mortality. This is likely due to the association between lifestyle-induced changes in waist circumference and cardiovascular risk factors. Reductions in waist circumference may be facilitated by diet without cereal grains combined with long-term physical exercise.

**Methods:**

Two-year randomised controlled trial with factorial trial design in individuals at increased risk of cardiovascular disease with increased waist circumference. Participants were allocated diet based on current Swedish dietary guidelines with or without cereal grains (baseline diet information supported by monthly group sessions) and with or without physical exercise (pedometers and two initial months of weekly structured exercise followed by written prescription of physical activity) or control group. The primary outcome was the change in waist circumference.

**Findings:**

The greatest mean intervention group difference in the change in waist circumference among the 73 participants (47 women and 26 men aged 23–79 years) was at one year between participants allocated a diet without cereal grains and no exercise and participants allocated a diet with cereal grains and no exercise [*M* = −5.3 cm and −0.9 cm, respectively; mean difference = 4.4 cm, 4.0%, 95% CI (0.0%, 8.0%), *P* = 0.051, Cohen’s d = 0.75]. All group comparisons in the change in waist circumference were non-significant despite the greatest group difference being more than double that estimated in the pre-study power calculation. The non-significance was likely caused by too few participants and a greater than expected variability in the change in waist circumference. The greatest mean intervention group difference strengthens the possibility that dietary exclusion of cereal grains could be related to greater reduction in waist circumference.

## Background

The proportion of adults with overweight or obesity increased worldwide between 1980 and 2013 from 29% to 37% in men and from 30% to 38% in women (Ng *et al*., 2014). Prevalence rates also increased substantially in children and among adolescents of both sexes in both developed and developing countries (Ng *et al*., 2014). Obesity and increased degree of obesity lead to increased risk of cardiovascular disease for both sexes (Piché *et al*., 2018). Risk also depends on body fat distribution, with increased risk when a greater proportion of body fat is stored abdominally (Piché *et al*., 2018). Elevated waist circumference is an indicator of increased abdominal fat storage and is accordingly associated with increased cardiovascular mortality (Piché *et al*., 2018; Ross *et al*., 2020). This is likely due to the association between lifestyle-induced changes in waist circumference and cardiovascular risk factors (Piché *et al*., 2018; Ross *et al*., 2020). Lifestyle-induced reductions in waist circumference can be induced by energy restriction (i.e., dietary caloric restriction) or an increase in energy expenditure (i.e., exercise) (Ross *et al*., 2020). Regarding energy restriction, waist circumference has, in randomised controlled trials (RCTs), decreased more from adopting a Palaeolithic diet based on fruits, vegetables, tubers, nuts, eggs, meat and fish at the expense of, among other food groups, cereal grains, compared to control diets (de Menezes *et al*., 2019). The exclusion of cereal grains with accompanying beneficial effects on satiety regulation could be related to this greater reduction in waist circumference (Jönsson *et al*., 2015, 2010). Regarding the combination of energy restriction with increases in energy expenditure, a short-term study among individuals with type 2 diabetes did not find a greater reduction in waist circumference from exercise combined with Palaeolithic diet compared to Palaeolithic diet alone (Otten *et al*., 2017). However, weight loss studies indicate that longer-term interventions could reveal an increased effect from combined diet and exercise compared to diet alone (Johns *et al*., 2014).

There is, thus, a need to study the effects on waist circumference, both from diets with or without cereal grains and from the combination of diets with or without long-term physical exercise. Both effects could efficiently be studied simultaneously using a factorial trial design. The rationale for using a factorial trial design is that it allows for the examination of main effects of two or more independent variables simultaneously. A disadvantage of a factorial trial design is the need for more participants – albeit fewer compared to conducting separate studies. This disadvantage can be mitigated by studying healthy participants at increased risk of cardiovascular disease in a primary health care setting. This would both improve the possibility of sufficient recruitment and aim preventative interventions towards those who stand to benefit the most in a setting with a preventive mission.

Our aim, therefore, using a long-term randomised controlled intervention study with a factorial trial design implemented in primary health care, was to assess the effects on waist circumference of a diet based on the Swedish Food Agency’s dietary guidelines with or without cereal grains and with or without physical exercise in individuals with increased waist circumference and at least one other cardiovascular risk factor.

## Material and methods

The Regional Ethical Board in Lund approved the study protocol (LU 2010/332), which adhered to the Declaration of Helsinki, and all participants gave written informed consent.

### Study design and population

**Figure 1.**
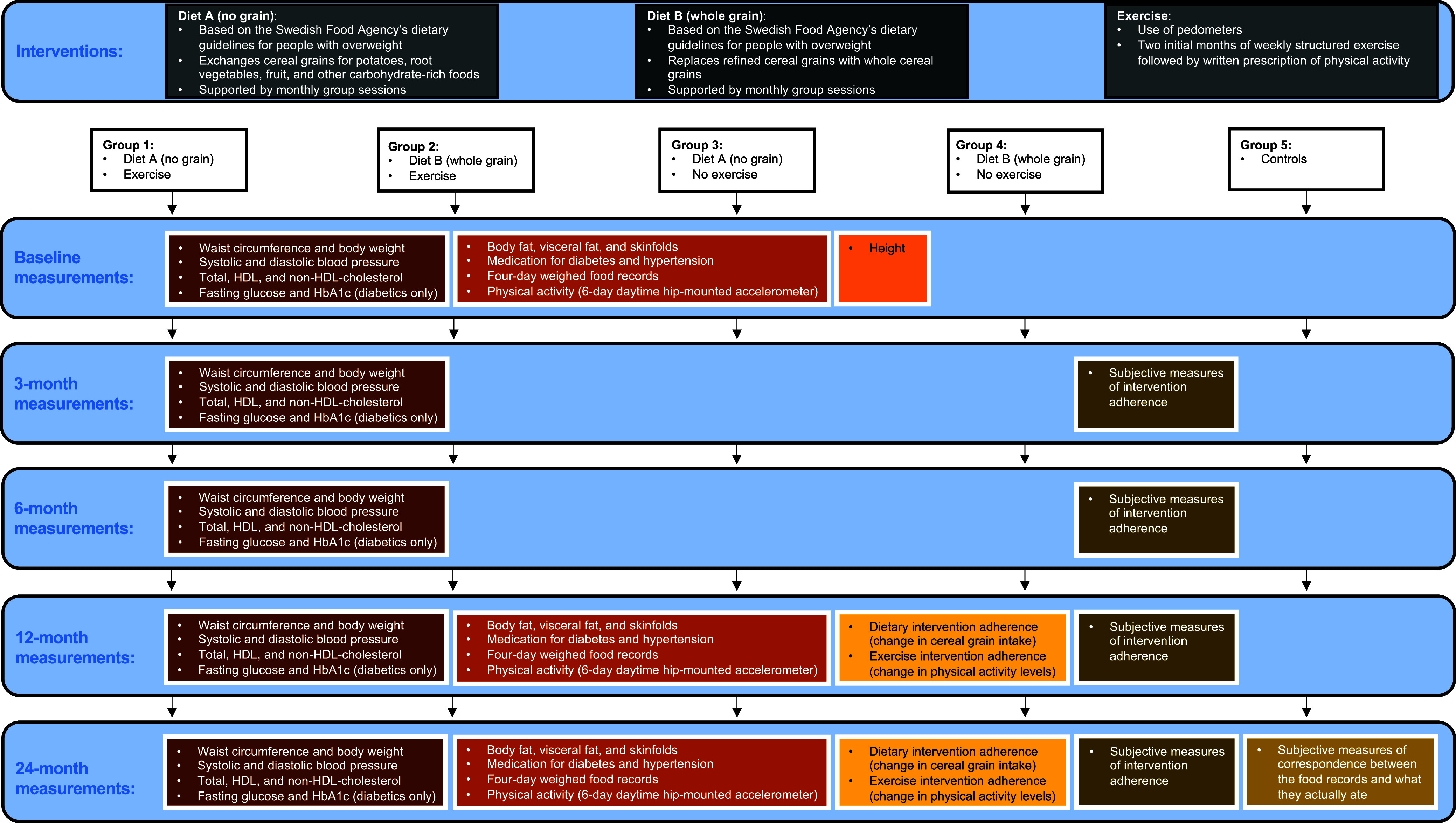
Study design with interventions and measurements during the study

This study is a two-year-long RCT with a factorial design conducted among adult participants with increased waist circumference and at least one additional risk factor for cardiovascular disease, who belonged to a public Swedish primary health care centre in the university town of Lund, Sweden. A study design overview with interventions and measurements is depicted in Figure 1. Participants were allocated to eat a healthy diet based on the Swedish Food Agency’s dietary guidelines for people affected by overweight with or without grains and with or without physical exercise or to a control group with follow-up only. The study started in August 2010 and ended in November 2014. The primary outcome was the change in waist circumference during two years. The secondary outcomes were changes in blood pressure, body fat, non-high-density lipoprotein (non-HDL) cholesterol, accelerometer physical activity, change in blood sugar-lowering medications and, for diabetics only, also fasting blood sugar and haemoglobin A1c (HbA1c) during two years. Potentially eligible participants were recruited from patients attending the primary health care centre and volunteering individuals belonging to the primary health care centre who heard of the study through leaflets distributed in the waiting room of the primary health care centre or via advertising in the local daily newspaper. Inclusion criteria were increased waist circumference (≥84 cm in women and ≥98 cm in men) and at least one of the following additional risk factors for cardiovascular disease: a history of coronary heart disease, stroke or transitory ischaemic attack, peripheral arterial disease, hypertension, type 2 diabetes, impaired glucose tolerance, prior gestational diabetes, smoking and first degree relative with cardiovascular disease before 60 years of age or with type 2 diabetes. The sex-specific cut-off points for increased waist circumference in this study were set halfway between the World Health Organization (WHO) example of cut-off points for increased and substantially increased risk of metabolic complications associated with obesity in Caucasians (World Health Organization, 2011). This choice of sex-specific cut-off points was made to ensure that participants would have an increased waist circumference that could decrease substantially during the study’s duration before their waist circumference became less than the cut-off point for increased risk of metabolic complications associated with obesity in Caucasians (World Health Organization, 2011). Exclusion criteria were gluten intolerance, dependence on walking aids, difficulty understanding oral or written Swedish language, age <20 years, body mass index >40 kg/m^2^, cognitive impairment, pronounced hearing loss, aphasia and continuous treatment with anticoagulants or oral cortisone medications.

Eligible participants were allocated via randomisation by a study nurse or doctor with the participant in the room using an Internet-based random number generator from the School of Computer Science and Statistics, Trinity College, Dublin (http://www.random.org), to one of the five groups with equal chance (20%) of being allocated to each group and were then followed up for two years: group 1 was allocated diet A and physical exercise; group 2 was allocated diet B and physical exercise; group 3 was allocated diet A; group 4 was allocated diet B and group 5 was allocated only follow-up and termed ‘controls’.

Both diets A and B were based on the Swedish Food Agency’s dietary guidelines for people with overweight (eat plenty of fruit, vegetables and fish, choose low-fat meat and low-fat dairy products and avoid candy, ice cream, snacks, cakes, pastries, chocolate, potato chips, beer, soft drinks and juice). The diets were based on the Swedish Food Agency’s dietary guidelines since these are based on available science, are independent of special interests and form the basis for dietary advice in Swedish health care (Swedish Food Agency, 2023). The diets differed from each other in that diet A, which was termed ‘no grain’, encouraged exchanging cereal grain products for potatoes, root vegetables, fruit and other carbohydrate-rich foods, while diet B, which was termed ‘whole grain’, encouraged replacing refined cereal grain products with whole cereal grain products, with the aim of keeping the carbohydrate content similar in the two diets. The dietary advice specific for diet A (no grain) stated that all types of grain should be avoided (bread, pasta, cereals, porridge, pies, crackers, whole grain and rice) and that they should eat (1) potatoes, root vegetables, corn and beans instead of pasta, rice and bread, (2) fruit instead of sandwiches and buns, (3) eggs/omelettes and fruit/fruit salad with sour milk/yogurt for breakfast, (4) fruit and nuts instead of cookies and candy and (5) fruit salad as dessert (with ice cream when it is a party). The dietary advice specific for diet B (whole grain) recommended replacing refined cereal grain products with whole grain cereal products (bread, muesli, semolina and pasta).

Directly upon randomisation, participants allocated dietary intervention received dietary information regarding their allocated diet individually both orally and in writing (when possible, together with spouse/cohabitant) and were also offered the opportunity to continuously ask questions regarding their allocated diet via email during the study. Participants allocated dietary intervention were also encouraged to attend voluntary monthly (except for July) 1 h group meetings held during the study period with information regarding their allocated diet. Directly upon randomisation, participants allocated physical exercise, termed ‘exercise’, received instructions on how to use purchased pedometers and were scheduled to participate in fee-based structured group training for increased cardiorespiratory fitness at the primary health care centre. The exercise, which was supervised by a certified physiotherapist, was for 2 h twice a week for four weeks and then once a week for another four weeks for the first eight weeks of the study. After these first eight weeks of supervised group training, the participants allocated physical exercise were encouraged, by the certified physiotherapists holding the group training sessions at the primary health care centre, to continue physical exercise training on their own for the rest of the study and received from them, for this purpose, a written prescription of suitable physical activity (Onerup *et al*., 2019). The allocation to no physical exercise was termed ‘no exercise’. All participants were encouraged to practice regular physical activity according to the recommendations and also recommended to use pedometers as well as offered physical activity on prescription as part of usual care.

## Measurements

Waist circumference was measured using a measuring tape with the patient in a standing position after breathing out. The measurement was made at the height of the lower edge of the elbow kept at 90° next to the body. The measurement was read in the middle axillary line with the measuring tape in a horizontal circle (World Health Organization, 2000). For waist circumference, a short-term 5% decrease and a long-term 3% decrease are both considered to be clinically relevant changes, and at least a short-term 5% decrease is considered detectable since the intra-observer measurement error is smaller than 4 cm in most studies (Verweij *et al*., 2013). Body weight was measured using the electronic weighing scale of the laboratory at the primary health care centre with the subjects in the fasting state with their weight evenly distributed between both feet placed in the centre of the platform and without shoes, jacket, sweater or other heavy clothing and without heavy items in their pockets. Standing height, without shoes, was measured with the same wall-mounted height stadiometer at the laboratory of the primary health care centre. Systolic and diastolic blood pressures were calculated from the mean of two measurements separated by 5 s at heart level on the upper arm with an automated device and the subjects sitting without previous rest (WHO Monica criteria) (WHO MONICA Project Principal Invest, 1988). The total body fat percentage was assessed by the use of a Tanita hand-foot bioelectrical impedance analysis system (Tanita BC-545), which has a 3%–5% accuracy compared with gold-standard methods (Ceniccola *et al*., 2019). The thickness of subcutaneous fat was assessed by the use of a calliper measuring skinfold at four sites (biceps, in the anterior part of the arm in the middle point between the most external and superior border of the acromion and the most external and superior of the radial bone head; triceps, in the posterior part of the arm in the middle point between the lower border of the acromion and the vertex of the olecranon; supra-iliac, approximately 2.5 cm above the iliac crest in the mid-axillary line; and subscapular, in the inferior vertex of the scapular bone). The correspondence between skinfold measurements and gold-standard methods in estimating subcutaneous fat is considered to be acceptable, provided the examiner is consistently the same and follows a standardised protocol, as was practised in this study (Fosbøl and Zerahn, 2015).

Blood samples were drawn in the morning at the laboratory of the primary health care centre with the subjects in an overnight fasting state and sent to the accredited central laboratory of Lund University Hospital for analyses of total and HDL cholesterol, fasting blood sugar and HbA1c. Non-HDL cholesterol was calculated by subtracting HDL cholesterol from total cholesterol. Physical activity was continuously monitored using a hip-mounted accelerometer (ActiGraph GT3X™, Actigraph Corporation, Pensacola, FL) worn daytime for six days. The ActiLife analysis software platform was used to achieve physical activity measures, summarising vertical axis data into 60 s epochs, excluding non-wear time determined as 60 min of zero and applying previously developed cut-off points to assess the time spent in different physical activity intensity levels (Freedson *et al*., 1998).

Food intake during the study was assessed from each subject’s reports of four-day weighed food records, including one weekend day, with weighing of each food item on an electronic weighing scale (which could be tared). Participants were thus instructed to, as far as possible, weigh every food item and if weighing was not possible, instead, enter quantities or measurements in other ways such as decilitres, tablespoons, teaspoons or centimetres. Although the latter units of measurement are less precise, this should be a minor weakness of the method since the large majority of food items in this and our previous studies were weighed and since the other measurements were translated into grams by YG, who is experienced in the translation of food records. In parallel with the four-day weighed food records, the subjects recorded their subjective rating of satiety before starting a meal and 30 min after starting the meal by means of a seven-point Likert scale going from ‘very hungry’ to ‘very full’, which has a high reliability across sessions and a moderate sensitivity for differentiating satiety effects of various foods (Merrill *et al*., 2002). Satiety quotients were calculated, as the intra-meal quotient of change in satiety during a meal and consumed energy or weight of food and drink for that specific meal. The four-day weighed food records with satiety ratings were recorded using paper diaries, which, although at greater risk of becoming lost or filled with illegible text, were deemed as less cumbersome for the participants compared to available digital and mobile solutions. Although all self-reporting of food intake is prone to under-reporting, the diaries were deemed by YG, who is experienced in the translation of food records, to be mostly adequately completed with many reports of singular intakes of fruits, snacks and drinks in between larger meals and comparatively as complete as dietary records in our previous studies (Lindeberg *et al*., 2007; Jönsson *et al*., 2009). Dietary nutrient content from reported food intake was calculated from the contemporary Food Database of the Swedish Food Agency, which was used since it is an updated, researcher independent and locally well-established food database with high credibility (Swedish Food Agency, 2011). Waist circumference, body weight, systolic and diastolic blood pressure, total and HDL cholesterol, fasting blood sugar and HbA1c were all measured at baseline and after 3, 6, 12 and 24 months. Standing height was measured at baseline. At 3, 6, 12 and 24 months, the subjects were asked to rate, on a scale from 0 to 10 with 10 being perfect compliance, their compliance with the allocated intervention from baseline to 3 months, 3–6 months, 6–12 months and 12–24 months, respectively. Body fat percentage, subcutaneous fat, physical activity, food intake and changes in medication for diabetes and hypertension were all measured and recorded at baseline and after 12 and 24 months. Upon completing their last four-day weighed food record of the study at 24 months, subjects were asked to rate, on a scale from 0 to 100 with 100 being perfect correspondence, how well the food records corresponded to how they actually ate during the study period.

Adherence to the dietary interventions was monitored and assessed with change in the intake of cereal grains and whole grains during the study extracted from four-day weighed food records at baseline, 12 and 24 months. Adherence to the exercise intervention was monitored and assessed with change in physical activity levels extracted from the hip-mounted accelerometer worn daytime for six days at baseline, 12 and 24 months.

## Statistical analysis

Statistical analysis was conducted using SPSS (version 28). Pre-study power calculation determined that 200 participants were needed for a standard two-sided two independent sample t-test to detect a 2 cm difference in waist circumference change (SD 2.9 cm, 20% drop-out) with 80% power and 5% significance level (Rosner, 2015). Assumptions for mean difference and SD were based on previous results (Lindeberg *et al*., 2007). Primary analysis was based on an intention-to-treat approach. Data normality was assessed with normal Q-Q plot and Shapiro–Wilk tests. Group comparisons were based on the χ^2^, Fisher exact, one-way analysis of variance (ANOVA) or Kruskal–Wallis tests, as appropriate, followed by Bonferroni-adjusted post hoc tests (χ^2^, Tukey, Mann–Whitney). Secondary analyses included mixed models and analysis of covariance. The significance was set at *P* < 0.05.

## Results

**Figure 2.**
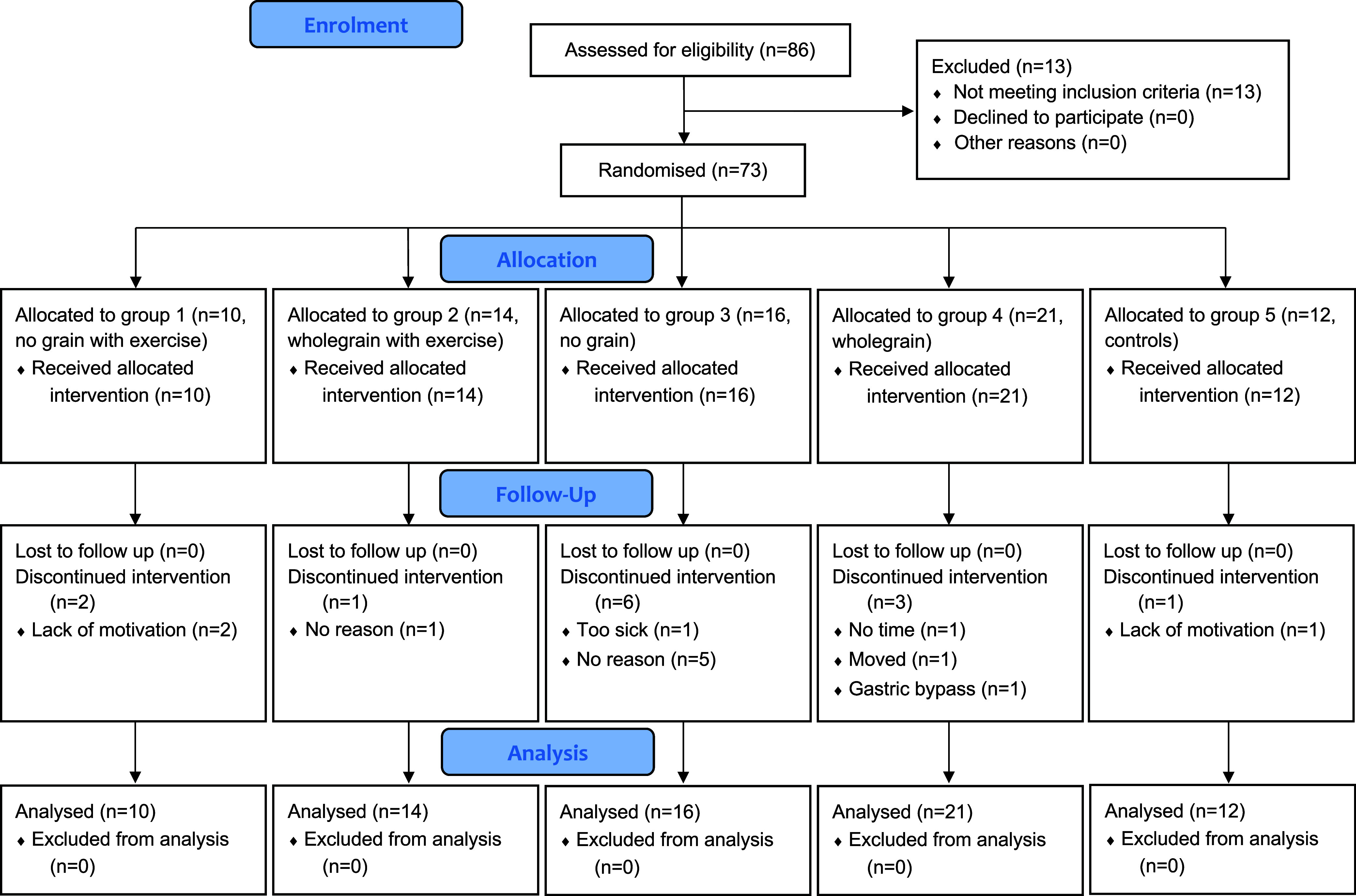
CONSORT diagram – flow of participants through study phases

Recruitment started in August 2010 and ended in November 2014 when recruitment had not yielded new participants for more than six months despite intensive efforts through leaflets distributed in the waiting room of the primary health care centre and advertisements in the local daily newspaper. We assessed 86 participants for eligibility, and 13 participants were excluded due to not meeting the inclusion criteria. We randomised 73 participants (47 women and 26 men aged 23–79 years), who started the trial immediately upon randomisation at different times of the year, although not during the winter holidays or in the summer holiday month of July. Discontinuation was most common after 12 months of participation among the 13 participants who discontinued the intervention. The study was completed in its entirety by 60 participants (37 women and 23 men). All 73 randomised participants were included in our primary analysis conducted using an intention-to-treat approach (Consolidated Standards of Reporting Trials (CONSORT) diagram in Fig. 2). There were no significant differences between groups in baseline characteristics, study discontinuation, medication and risk factors for cardiovascular disease (Tables 1 and 2). There were no reported harms to the participants derived from the interventions at any time point of the trial.

**Table 1.** Baseline characteristics

	Group 1		Group 2		Group 3		Group 4		Group 5				
	Exercise				No exercise						One-way ANOVA		
Variable	Diet A (no grain)		Diet B (whole grain)		Diet A (no grain)		Diet B (whole grain)		Controls		*F*(4, 61–68^a^)	η^*2*^	*P*
Participants, *n*	10		14		16		21		12				
Male/female, *n*	3/7		2/12		6/10		10/11		5/7				0.35
Age, years *M (SD)*	59	(11)	64	(5)	56	(13)	58	(12)	62	(8)	1.30	0.07	0.28
Height, cm *M (SD)*	168	(11)	169	(9)	170	(11)	174	(9)	169	(7)			0.40
Waist circumference, cm *M (SD)*	109	(11)	106	(11)	110	(12)	106	(11)	108	(14)			0.80
Weight, kg *M (SD)*	95	(16)	93	(14)	95	(14)	93	(14)	91	(17)	0.17	0.01	0.95
Body mass index, kg/m^2^ *M (SD)*	33	(3)	33	(5)	33	(6)	31	(3)	31	(5)			0.38
Systolic blood pressure, mm Hg *M (SD)*	136	(21)	154	(16)	139	(17)	146	(17)	143	(20)			0.17
Diastolic blood pressure, mm Hg *M (SD)*	84	(13)	84	(9)	84	(13)	87	(8)	82	(7)			0.70
Fasting plasma glucose, mmol/L *M (SD)*	8.3	(1.3)	7.6	(0.5)	7.5	(0.9)	8.8	(3.2)	8.8	(1.3)	0.47	0.13	0.76
HbA1C, mmol/mol *M (SD)*	50	(3)	42	(2)	54	(11)	50	(18)	49	(12)			0.34
Total cholesterol, mmol/L *M (SD)*	4.9	(0.8)	5.3	(1.2)	5.2	(1.2)	5.5	(1.3)	5.0	(1.2)	0.68	0.04	0.61
HDL, mmol/L *M (SD)*	1.2	(0.2)	1.6	(0.5)	1.2	(0.3)	1.2	(0.3)	1.3	(0.4)			0.059
Non-HDL, mmol/L *M (SD)*	3.8	(0.9)	3.8	(1.2)	4.0	(1.1)	4.3	(1.2)	3.7	(1.0)	0.87	0.05	0.48
Body fat, % *M (SD)*	43_b_	(6)	43	(6)	36	(8)	35	(8)	36_b_	(4)			0.011
Visceral fat, % *M (SD)*	15	(5)	13	(3)	12	(4)	13	(4)	14	(6)	0.71	0.05	0.59
Biceps skinfold, mm *M (SD)*	20	(5)	21	(9)	16	(6)	16	(6)	18	(7)	1.69	0.10	0.163
Triceps skinfold, mm *M (SD)*	32	(5)	31	(9)	27	(12)	26	(9)	26	(9)	1.29	0.08	0.28
Subscapular skinfold, mm *M (SD)*	38	(11)	34	(8)	33	(11)	33	(9)	35	(5)			0.31
Supra-iliac skinfold, mm *M (SD)*	31	(6)	27	(7)	27	(9)	26	(10)	27	(8)	0.52	0.03	0.72

*Note*. Means in a row sharing subscripts are significantly different from one another. HbA1C = haemoglobin A1C; HDL = high-density lipoprotein.

a 13 for fasting plasma glucose.

**Table 2.** Study discontinuation, medication and risk factors for cardiovascular disease

	Group 1	Group 2	Group 3	Group 4	Group 5	
	Exercise		No exercise			
Variable	Diet A (no grain)	Diet B (whole grain)	Diet A (no grain)	Diet B (whole grain)	Controls	*p*
Discontinued intervention no/yes, *n*	8/2	13/1	10/6	18/3	11/1	0.23
Medicine against hypertension,^a^ *n* none/reduced/unchanged/increased	2/0/7/1	3/1/6/4	7/2/7/0	5/1/11/4	7/0/5/0	0.21
Medicine against diabetes,^a^ *n* none/reduced/unchanged/increased	7/0/0/1	12/1/0/1	12/0/1/3	15/1/0/3	9/0/0/2	0.98
Ischaemic heart disease no/yes, *n*	10/0	12/2	15/1	17/4	11/1	0.64
Stroke or TIA no/yes, *n*	10/0	14/0	15/1	20/1	12/0	0.99
Peripheral arterial disease no/yes, *n*	10/0	14/0	16/0	21/0	12/0	0.99
Hypertension no/yes, *n*	2/8	2/12	7/9	5/16	5/7	0.33
Diabetes type 2 no/yes, *n*	7/3	11/3	11/5	15/6	8/4	0.97
IGT or pregnancy diabetes no/yes, *n*	10/0	14/0	14/2	21/0	11/1	0.23
Smoker no/yes, *n*	10/0	11/3	14/2	19/2	11/1	0.70
Heredity cardiovascular disease no/yes, *n*	7/3	11/3	12/4	11/10	10/2	0.36

*Note.* TIA = transient ischaemic attack; IGT = impaired glucose tolerance.

a Occurrence and change during study.

There were no significant differences between participants based on group, exercise or diet allocation in the primary ANOVA in change in the primary outcome waist circumference (Tables 3 and 4). In secondary analyses of the change in waist circumference, there were also no significant differences between participants for group, diet or exercise allocation in either ANOVA with or without sex as a fixed factor and with age and/or body fat percentage at baseline as covariate(s) or in the ANOVA per protocol, that is, in all participants who completed the study in its entirety.

**Table 3.** Outcome change from baseline

		Group 1		Group 2		Group 3		Group 4		Group 5				
		Exercise				No exercise								
		Diet A (no grain)		Diet B (whole grain)		Diet A (no grain)		Diet B (whole grain)		Controls		One-way ANOVA		
Variable	Time (months)	*M*	*(SD)*	*M*	*(SD)*	*M*	*(SD)*	*M*	*(SD)*	*M*	*(SD)*	*F*(4, 47–68^a^)	η^*2*^	*p*
Waist circumference	3	−2.8%	(3.7%)	−3.6%	(4.5%)	−4.4%	(4.6%)	−2.3%	(3.2%)	−2.1%	(3.3%)	0.83	0.05	0.51
	6	−2.7%	(7.4%)	−3.5%	(7.5%)	−4.4%	(5.5%)	−2.3%	(5.4%)	−4.1%	(3.3%)			0.65
	12	−1.7%	(7.6%)	−2.2%	(8.4%)	−4.7%	(5.3%)	−0.7%	(5.4%)	−3.6%	(4.0%)			0.22
	24	2.7%	(7.0%)	−0.5%	(6.6%)	−1.7%	(4.9%)	−0.6%	(8.0%)	−1.6%	(4.6%)	0.63	0.04	0.64
Weight	3	−2.1%	(4.1%)	−2.6%	(3.5%)	−3.1%	(3.9%)	−1.1%	(3.1%)	−1.1%	(4.2%)			0.28
	6	−1.7%	(5.7%)	−3.0%	(3.6%)	−3.8%	(5.3%)	−1.6%	(4.3%)	−2.7%	(5.6%)			0.46
	12	−2.1%	(5.3%)	−2.5%	(4.4%)	−3.1%	(6.6%)	−0.6%	(4.6%)	−2.1%	(5.5%)			0.53
	24	−2.0%	(5.0%)	−2.6%	(4.2%)	−1.6%	(6.4%)	−1.8%	(5.1%)	−1.0%	(5.1%)			0.77
Systolic blood pressure	3	1.3%	(16.6%)	−8.3%	(10.1%)	−8.4%	(12.1%)	−4.8%	(10.2%)	−1.1%	(10.3%)			0.47
	6	−0.4%	(13.6%)	−7.0%	(12.0%)	−4.3%	(7.5%)	−2.2%	(13.2%)	−2.4%	(12.1%)			0.84
	12	−2.4%	(12.9%)	−6.6%	(6.6%)	−2.6%	(8.5%)	−3.5%	(11.2%)	−4.3%	(9.7%)			0.66
	24	−4.5%	(12.1%)	−4.5%	(11.7%)	0.6%	(5.6%)	−4.3%	(11.4%)	1.7%	(11.7%)	0.97	0.07	0.43
Diastolic blood pressure	3	4.3%	(16.9%)	−5.4%	(8.7%)	−6.4%	(10.5%)	−3.9%	(9.6%)	−3.0%	(9.1%)			0.37
	6	1.4%	(11.6%)	−5.6%	(12.2%)	−3.8%	(9.8%)	−4.2%	(9.4%)	−3.2%	(8.4%)	0.69	0.04	0.60
	12	−4.4%	(14.9%)	−6.7%	(5.8%)	−4.1%	(7.3%)	−2.2%	(8.0%)	−3.2%	(8.0%)	0.59	0.04	0.67
	24	−6.4%	(13.1%)	−2.4%	(8.7%)	−0.5%	(7.7%)	−4.6%	(8.9%)	1.7%	(6.9%)			0.28
Fasting plasma glucose	3	4.0%	(1.6%)	−9.0%	(8.8%)	1.9%	(22.7%)	14.9%	(9.2%)	17.5%	(42.7%)			0.41
	6	14.7%	(11.6%)	−15.4%	(15.9%)	−10.6%	(13.0%)	−11.9%	(25.5%)	−9.7%	(28.3%)	1.03	0.27	0.43
	12	6.1%	(17.0%)	−9.7%	(5.9%)	−7.7%	(24.9%)	−18.9%	(26.3%)	−11.1%	(24.4%)	0.53	0.16	0.72
	24	−10.8%	(3.8%)	−12.7%	(0.0%)	−12.1%	(9.2%)	−18.0%	(21.4%)	−18.1%	(20.6%)			0.97
HbA1C	3	4.0%	(0.3%)	−0.8%	(3.6%)	−5.5%	(13.3%)	25.8%	(23.7%)	2.8%	(45.7%)			0.21
	6	7.9%	(8.3%)	−3.0%	(9.5%)	−4.9%	(16.8%)	15.3%	(28.0%)	−0.4%	(33.1%)			0.66
	12	8.0%	(2.0%)	0.2%	(6.5%)	1.3%	(27.1%)	−5.4%	(27.3%)	2.6%	(33.0%)	0.13	0.04	0.97
	24	−3.5%	(3.3%)	4.6%	(6.3%)	−1.9%	(17.1%)	−7.2%	(15.0%)	5.4%	(36.8%)	0.25	0.07	0.91
Total cholesterol	3	7.2%	(13.8%)	−4.0%	(8.3%)	−1.5%	(13.7%)	−3.7%	(7.9%)	2.3%	(17.5%)	1.73	0.10	0.154
	6	5.9%	(16.7%)	−0.2%	(9.7%)	−0.7%	(11.1%)	−2.9%	(11.6%)	−3.6%	(15.0%)			0.61
	12	4.4%	(19.8%)	3.8%	(10.5%)	−0.9%	(14.0%)	−3.3%	(12.8%)	1.7%	(10.1%)			0.80
	24	−5.8%	(7.3%)	0.5%	(11.0%)	0.3%	(15.6%)	−7.8%	(11.5%)	−0.9%	(13.5%)	1.33	0.09	0.27
HDL	3	8.2%	(20.1%)	−3.9%	(11.9%)	3.3%	(10.3%)	5.9%	(15.2%)	6.8%	(11.1%)			0.25
	6	7.5%	(19.8%)	5.0%	(15.5%)	4.3%	(19.4%)	11.4%	(11.0%)	8.1%	(10.5%)			0.52
	12	14.1%	(20.7%)	3.3%	(12.0%)	27.1%	(61.3%)	4.8%	(13.1%)	7.4%	(9.6%)			0.56
	24	4.2%	(14.0%)	15.2%	(44.3%)	8.6%	(17.9%)	6.6%	(11.7%)	4.9%	(14.5%)			0.97
Non-HDL	3	7.8%	(18.1%)	−6.7%	(12.4%)	−2.8%	(17.7%)	−5.9%	(8.3%)	1.1%	(21.0%)			0.166
	6	6.7%	(22.3%)	−2.4%	(12.6%)	−2.3%	(15.6%)	−6.6%	(14.1%)	−6.7%	(19.1%)			0.55
	12	0.5%	(29.3%)	1.9%	(14.1%)	−8.9%	(17.8%)	−4.6%	(16.6%)	−0.3%	(14.7%)			0.63
	24	−8.1%	(7.7%)	−7.3%	(18.7%)	−2.3%	(18.7%)	−12.0%	(13.7%)	−2.6%	(16.6%)			0.42
Body fat	12	−3.1%	(6.3%)	−0.8%	(4.9%)	−0.3%	(7.7%)	−1.0%	(16.5%)	0.4%	(10.4%)			0.52
	24	−2.4%	(5.8%)	−1.1%	(4.3%)	4.6%	(5.0%)	0.6%	(11.6%)	0.7%	(7.7%)			0.24
Visceral fat	12	−2.0%	(12.3%)	−2.1%	(6.7%)	1.5%	(14.5%)	3.8%	(9.3%)	2.2%	(11.6%)			0.23
	24	0.9%	(12.1%)	−2.9%	(7.4%)	4.8%	(5.3%)	1.2%	(12.6%)	2.3%	(11.8%)			0.30
Biceps skinfold	12	−3.7%	(21.9%)	−1.5%	(24.2%)	−2.9%	(30.7%)	17.6%	(35.2%)	1.1%	(25.7%)			0.25
	24	2.2%	(21.8%)	−5.3%	(25.8%)	−5.6%	(21.6%)	2.3%	(23.8%)	−1.7%	(18.9%)	0.35	0.03	0.85
Triceps skinfold	12	−6.4%	(18.5%)	9.7%	(29.8%)	7.9%	(25.0%)	4.4%	(15.4%)	−7.5%	(14.9%)			0.190
	24	−5.3%	(15.9%)	11.8%	(30.2%)	5.3%	(29.3%)	12.2%	(39.6%)	4.6%	(25.5%)			0.74
Subscapular skinfold	12	−2.4%	(15.9%)	0.3%	(20.7%)	13.1%	(51.3%)	7.5%	(27.2%)	−6.5%	(16.2%)			0.60
	24	−2.4%	(19.4%)	3.3%	(14.1%)	24.7%	(52.0%)	10.6%	(27.7%)	−1.7%	(15.8%)			0.52
Supra-iliac skinfold	12	0.5%	(27.0%)	8.3%	(20.9%)	7.8%	(26.1%)	1.2%	(24.4%)	3.3%	(14.9%)			0.90
	24	−3.9%	(25.3%)	9.0%	(32.0%)	4.5%	(26.9%)	11.5%	(29.9%)	13.1%	(22.7%)	0.51	0.04	0.73

*Note.* ANOVA = analysis of variance; HDL = high-density lipoprotein cholesterol; HbA1C = haemoglobin A1C.

a 8–18 for glucose and HbA1C.

**Table 4.** Between group comparisons of change from baseline in waist circumference

Group	Time (months)	Group 1				Group 2				Group 3				Group 4			
		Diet A (no grain) + exercise				Diet B (whole grain) + exercise				Diet A (no grain) + no exercise				Diet B (whole grain) + no exercise			
		Mean difference	95% CI of the difference		*P*	Mean difference	95% CI of the difference		*P*	Mean difference	95% CI of the difference		*P*	Mean difference	95% CI of the difference		*P*
			Lower	Upper			Lower	Upper			Lower	Upper			Lower	Upper	
2	3	0.8%	−2.9%	4.5%	0.66												
3	3	1.6%	−2.2%	5.3%	0.39	0.8%	−2.7%	4.3%	0.64								
4	3	−0.5%	−3.2%	2.3%	0.72	−1.3%	−3.9%	1.4%	0.33	−2.1%	−4.7%	0.6%	0.122				
5 (controls)	3	−0.6%	−4.0%	2.7%	0.70	−1.4%	−4.9%	2.0%	0.40	−2.2%	−5.7%	1.3%	0.20	−0.2%	−2.7%	2.4%	0.90
2	6	0.8%			0.31												
3	6	1.7%			0.49	0.9%			0.90								
4	6	−0.4%			0.84	−1.2%			0.30	−2.1%	−5.8%	1.6%	0.26				
5 (controls)	6	1.4%			0.40	0.6%			0.32	−0.3%			0.82	1.8%	−1.8%	5.5%	0.34
2	12	0.5%			0.41												
3	12	3.0%	−2.9%	8.9%	0.30	2.5%			0.64								
4	12	−1.0%	−6.1%	4.1%	0.69	−1.5%			0.084	−4.0%	−8.1%	0.0%	0.051				
5 (controls)	12	1.9%	−3.7%	7.4%	0.49	1.3%			0.66	−1.2%	−5.3%	3.0%	0.56	2.8%	−1.0%	6.7%	0.139
2	24	3.2%	−3.1%	9.6%	0.31												
3	24	4.4%	−1.5%	10.3%	0.135	1.2%	−4.0%	6.3%	0.65								
4	24	3.4%	−3.3%	10.0%	0.31	0.1%	−5.3%	5.6%	0.96	−1.0%	−6.7%	4.7%	0.72				
5 (controls)	24	4.3%	−1.3%	9.9%	0.123	1.1%	−3.9%	6.0%	0.66	−0.1%	−4.4%	4.2%	0.97	0.9%	−4.5%	6.3%	0.73

*Note.* The greatest intervention group difference in the change in waist circumference was at one year between group 3 (*M* = −5.3 cm = −4.7%, *SD* = 5.3%) and group 4 (*M* = −0.9 cm = −0.7%, *SD* = 5.4%) with a non-significant group difference [mean difference = 4.4 cm = 4.0%, 95% CI (0.0%, 8.0%), *t*(29) = −2.037, *p* = 0.051, Cohen’s d = 0.75].

The greatest intervention group difference in the change in waist circumference was at one year between participants allocated a diet without cereal grains and no physical exercise (*M* = −5.3 cm = −4.7%, *SD* = 5.3%) and participants allocated a diet with cereal grains and no physical exercise (*M* = −0.9 cm = −0.7%, *SD* = 5.4%) with a non-significant group difference [mean difference = 4.4 cm = 4.0%, 95% CI (0.0%, 8.0%), *t*(29) = −2.037, *P* = 0.051, Cohen’s d = 0.75] (Table 4).

**Figure 3.**
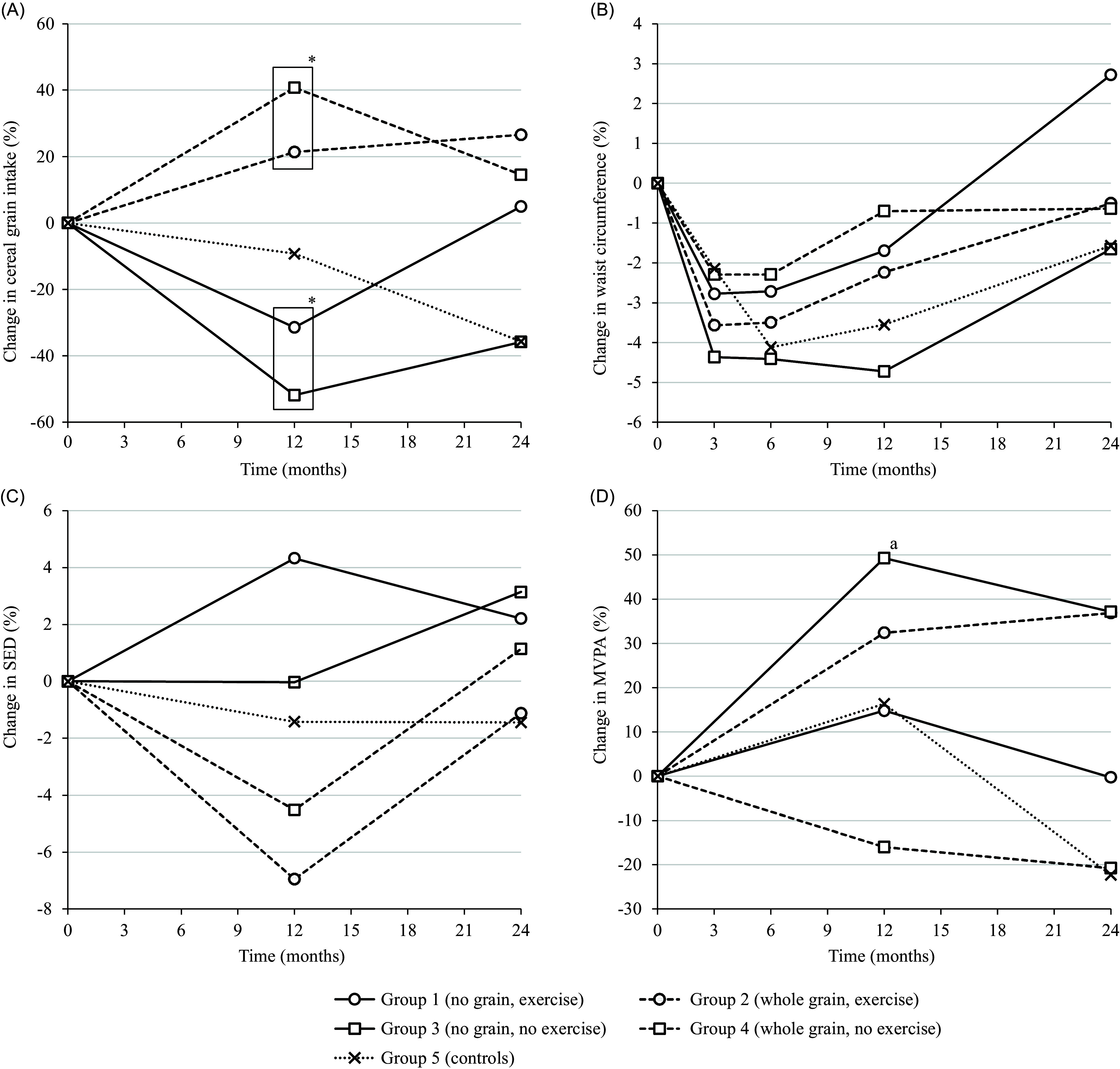
Intervention adherence *Note.* Group changes in intervention adherence and waist circumference. Error bars not included for clarity. SED = sedentary time. MVPA = moderate and vigorous physical activity. ^a^Without two extreme outliers. * *P* < 0.001 for difference in pooled means between groups 1+3 and 2+4. There were no other significant differences between groups in the presented figures.

A mixed model analysis found a (quadratic) trend over time for an initial decrease in waist circumference with the greatest reductions at 6 and 12 months followed by a return towards baseline values at the end of the study at 24 months (Fig. 3) – the trend was detected among participants based on group, exercise and diet allocation. The mixed model analysis otherwise found no significant differences in linear change over time in waist circumference between participants based on group, exercise or diet allocation, except for a difference between group 1 (no grain with exercise) and group 5 (controls) (*P* = 0.02), with less reduction at 24 months in group 1 (no grain with exercise) compared to group 5 (controls) (*P* = 0.03).

Primary analysis of secondary outcomes and dietary intakes is presented in Tables 2, 3 and 5–7. In sensitivity tests, with the exclusion of participants with fewer than six days of accelerometer physical activity measurements at baseline, 12 months or 24 months, there were also no significant differences between participants based on exercise allocation for any measures of accelerometer physical activity.

**Table 5.** Dietary intervention adherence and waist circumference

Variable	Time (months)	Group 1+3		Group 2+4		Group 5		One-way ANOVA		*P*
		Diet A (no grain)		Diet B (whole grain)		Controls		*F*(2, 58–70)	η^*2*^	
		*M*	(SD)	*M*	(SD)	*M*	(SD)			
Cereal grains intake, g	0	249	(153)	185	(100)	243	(113)			0.22
Cereal grains intake, change from baseline	12	−43%_a_	(66%)	28%_a_	(84%)	−9%	(59%)			<0.001
	24	−17%	(55%)	12%	(96%)	−36%	(24%)			0.122
Whole grain intake, g	0	40	(31)	36	(22)	35	(27)			0.94
Whole grain intake, change from baseline	12	−32%_a_	(96%)	98%_a_	(201%)	−17%	(61%)			<0.001
	24	29%	(96%)	62%	(155%)	14%	(103%)			0.56
Carbohydrate intake, g	0	207	(55)	198	(72)	226	(52)	0.78	0.03	0.46
Carbohydrate intake, change from baseline	12	−11%	(38%)	−5%	(32%)	−17%	(15%)			0.38
	24	−8%	(42%)	−8%	(37%)	−22%	(13%)			0.82
Waist circumference, cm	0	110	(11)	106	(10)	108	(14)	0.81	0.02	0.45
Waist circumference, change from baseline	3	−3.8%	(4.3%)	−2.8%	(3.8%)	−2.1%	(3.3%)	0.75	0.02	0.48
	6	−3.8%	(6.2%)	−2.8%	(6.2%)	−4.1%	(3.3%)			0.96
	12	−3.4%	(6.4%)	−1.4%	(6.8%)	−3.6%	(4.0%)			0.68
	24	0.3%	(6.1%)	−0.6%	(7.3%)	−1.6%	(4.6%)	0.28	0.01	0.76
Subjective rating of correspondence between diet registration and intake	24	64%	(35%)	72%	(28%)	89%	(11%)			0.160
Subjective compliance rating, units (0–10)	0–3	7.8	(2.8)	7.0	(2.2)	5.7	(0.6)			0.134
	3–6	7.2	(3.3)	6.3	(2.0)	6.0	(0.0)			0.161
	6–12	5.3	(3.2)	5.7	(2.3)	6.3	(0.6)			0.95
	12–24	3.8	(2.7)	5.6	(2.4)	6.3	(0.6)			0.061

*Note.* Means in a row sharing subscripts are significantly different from one another. ANOVA = analysis of variance.

**Table 6.** Exercise intervention adherence and waist circumference

Variable	Time (months)	Group 1+2		Group 3+4		Group 5		One-way ANOVA		*P*
		Exercise		No exercise		Controls		*F*(2, 45–70)	η^*2*^	
		*M*	*(SD)*	*M*	*(SD)*	*M*	*(SD)*			
SED, min/d	0	564	(75)	602	(91)	621	(56)	2.47	0.07	0.092
SED, change from baseline	12	−3%	(13%)	−3%	(12%)	−1%	(10%)	0.08	0.00	0.93
	24	0%	(10%)	2%	(17%)	−1%	(9%)	0.24	0.01	0.79
MVPA, min/d	0	23	(19)	27	(18)	29	(18)			0.45
MVPA, change from baseline	12	27%	(108%)	133%	(527%)	16%	(86%)			0.98
	12^a^			9%	(88%)					0.86
	24	23%	(100%)	3%	(102%)	−22%	(37%)			0.35
Waist circumference, cm	0	108	(11)	107	(11)	108	(14)			0.84
Waist circumference, change from baseline	3	−3.3%	(4.1%)	−3.2%	(3.9%)	−2.1%	(3.3%)			0.64
	6	−3.2%	(7.3%)	−3.2%	(5.4%)	−4.1%	(3.3%)			0.86
	12	−2.0%	(8.0%)	−2.3%	(5.6%)	−3.6%	(4.0%)	0.23	0.01	0.80
	24	0.7%	(6.8%)	−1.0%	(7.0%)	−1.6%	(4.6%)	0.59	0.02	0.56
Subjective compliance rating, units (0–10)	0–3	7.9	(1.9)	6.8	(2.7)	5.7	(0.6)			0.100
	3–6	7.4	(1.8)	6.1	(2.9)	6.0	(0.0)			0.28
	6–12	6.2	(2.2)	5.1	(2.8)	6.3	(0.6)			0.45
	12–24	5.2	(2.0)	4.8	(3.1)	6.3	(0.6)			0.70

*Note.* ANOVA = analysis of variance; SED = sedentary time; MVPA = moderate and vigorous physical activity.

a Without two extreme outliers in group 3.

**Table 7.** Dietary intake

Variable	Time (months)	Group 1+3		Group 2+4		Group 5		One-way ANOVA		*P*
		Diet A (no grain)		Diet B (whole grain)		Controls		*F*(2, 54–61)	η^*2*^	
		*M*	*(SD)*	*M*	*(SD)*	*M*	*(SD)*			
Weight, g *M (SD)*	0	2662	(766)	2719	(760)	2410	(638)	0.71	0.02	0.50
	12	2273	(641)	2462	(784)	2296	(446)	0.53	0.02	0.59
	24	2102	(472)	2227	(784)	2039	(511)			0.72
Energy, kJ *M (SD)*	0	8098	(1698)	7709	(2400)	8613	(1389)			0.135
	12	7142	(2116)	6922	(2394)	7536	(1695)			0.46
	24	6635	(1311)	6406	(1985)	7455	(2091)	1.30	0.05	0.28
Energy, kcal *M (SD)*	0	1935	(406)	1842	(574)	2059	(333)			0.135
	12	1707	(506)	1654	(572)	1801	(406)			0.46
	24	1586	(314)	1531	(474)	1782	(500)	1.30	0.05	0.28
Protein, g *M (SD)*	0	82	(21)	78	(22)	84	(14)	0.47	0.02	0.63
	12	83	(25)	76	(26)	78	(19)	0.55	0.02	0.58
	24	74	(14)	68	(23)	79	(19)	1.26	0.04	0.29
Fat, g *M (SD)*	0	79	(23)	72	(29)	79	(19)			0.199
	12	68	(27)	62	(30)	72	(21)			0.36
	24	62	(19)	56	(25)	75	(31)			0.115
Carbohydrate, g *M (SD)*	0	207	(55)	198	(72)	226	(52)	0.78	0.03	0.46
	12	172	(55)	177	(62)	186	(56)	0.19	0.01	0.83
	24	172	(52)	167	(52)	176	(44)	0.13	0.00	0.88
Fibre, g *M (SD)*	0	21	(7)	21	(8)	22	(9)			0.93
	12	18	(7)	21	(8)	21	(5)	1.50	0.05	0.23
	24	18	(5)	20	(7)	21	(5)			0.37
Salt, g *M (SD)*	0	8	(3)	7	(3)	7	(2)	1.00	0.03	0.37
	12	7	(3)	7	(3)	7	(2)			0.66
	24	7	(3)	6	(2)	7	(2)			0.64
Ash, g *M (SD)*	0	18	(4)	17	(5)	17	(4)	0.54	0.02	0.58
	12	17	(5)	16	(6)	16	(3)			0.50
	24	15	(4)	15	(5)	16	(3)			0.28
Water, g *M (SD)*	0	2252	(761)	2326	(705)	1975	(583)	1.02	0.03	0.37
	12	1911	(612)	2105	(720)	1915	(414)	0.69	0.02	0.51
	24	1759	(448)	1895	(720)	1667	(488)	0.64	0.02	0.53
Alcohol, g *M (SD)*	0	4	(6)	7	(9)	8	(9)			0.169
	12	5	(8)	6	(8)	8	(7)			0.36
	24	0_ab_	(2)	6_a_	(8)	6_b_	(9)			0.0071
Monosaccharides, g *M (SD)*	0	32	(17)	34	(16)	39	(16)			0.44
	12	33	(14)	29	(15)	35	(22)	0.72	0.02	0.49
	24	31	(18)	27	(13)	34	(19)			0.53
Disaccharides, g *M (SD)*	0	41	(19)	44	(26)	49	(13)			0.30
	12	44	(20)	37	(20)	42	(17)			0.29
	24	36	(22)	35	(16)	36	(8)			0.70
Sucrose, g *M (SD)*	0	28	(15)	29	(21)	36	(11)			0.119
	12	29	(15)	24	(17)	30	(14)			0.142
	24	24	(16)	23	(13)	23	(6)			0.76
Whole grain, g *M (SD)*	0	40	(31)	36	(22)	35	(27)			0.94
	12	21_a_	(27)	52_a_	(35)	30	(26)			0.0022
	24	29	(15)	43	(32)	36	(38)			0.29
Sum of saturated fatty acids, g *M (SD)*	0	31	(11)	28	(13)	29	(9)			0.49
	12	25	(11)	23	(14)	24	(10)			0.35
	24	23	(7)	21	(12)	25	(12)			0.30
Fatty acids C4:0–C10:0, g *M (SD)*	0	2.4	(1.3)	2.2	(1.2)	2.2	(1.3)	0.21	0.01	0.81
	12	1.7	(1.0)	1.8	(1.5)	1.8	(0.9)			0.66
	24	1.9	(0.8)	1.7	(1.3)	1.7	(1.0)			0.39
Lauric acid C12:0, g *M (SD)*	0	1.6	(1.5)	1.3	(0.9)	2.0	(1.6)			0.61
	12	1.0	(1.0)	1.0	(0.8)	1.2	(0.7)			0.39
	24	1.0	(0.5)	1.0	(0.6)	1.0	(0.6)	0.004	0.00	0.99
Myristic acid C14:0, g *M (SD)*	0	3.3	(1.5)	2.9	(1.4)	2.9	(1.4)			0.53
	12	2.6	(1.2)	2.4	(1.7)	2.6	(1.2)			0.35
	24	2.5	(0.9)	2.2	(1.5)	2.4	(1.1)			0.34
Palmitic acid C16:0, g *M (SD)*	0	16	(5)	14	(7)	15	(5)			0.50
	12	13	(6)	12	(7)	13	(5)			0.40
	24	12	(4)	11	(6)	13	(6)			0.28
Stearic acid C18:0, g *M (SD)*	0	6.7	(2.7)	6.3	(3.4)	6.2	(1.7)			0.63
	12	6.0	(3.6)	5.1	(3.4)	4.8	(2.2)			0.25
	24	5.0	(1.8)	4.5	(2.6)	5.8	(2.9)			0.26
Arachidic acid C20:0, g *M (SD)*	0	0.16	(0.10)	0.13	(0.11)	0.14	(0.05)			0.14
	12	0.13	(0.09)	0.11	(0.09)	0.11	(0.07)			0.64
	24	0.08	(0.04)	0.09	(0.09)	0.15	(0.12)			0.46
Sum of monounsaturated fatty acids, g *M (SD)*	0	30	(10)	27	(11)	29	(8)			0.193
	12	26	(11)	23	(12)	27	(9)			0.33
	24	23	(8)	21	(9)	30	(15)			0.162
Palmitoleic acid C16:1, g *M (SD)*	0	1.4	(0.7)	1.2	(0.6)	1.2	(0.3)			0.179
	12	1.3	(0.7)	1.1	(0.6)	1.2	(0.5)			0.30
	24	1.1	(0.5)	0.9	(0.5)	1.2	(0.4)			0.134
Oleic acid C18:1, g *M (SD)*	0	27	(10)	24	(10)	27	(8)			0.23
	12	24	(11)	21	(11)	25	(8)			0.35
	24	21	(8)	19	(9)	27	(15)			0.22
Sum of polyunsaturated fatty acids, g *M (SD)*	0	11	(3)	11	(4)	14	(6)			0.25
	12	10	(6)	10	(6)	14	(4)			0.056
	24	10	(4)	9	(4)	15	(7)			0.096
Linoleic acid C18:2, g *M (SD)*	0	8.0	(3.0)	7.6	(3.6)	10.6	(5.9)			0.169
	12	7.2_a_	(4.5)	7.5	(4.5)	10.3_a_	(3.7)			0.024
	24	7.2	(3.1)	6.7	(3.2)	10.6	(6.2)			0.23
Arachidonic acid C20:4, g *M (SD)*	0	0.13	(0.07)	0.11	(0.08)	0.11	(0.05)			0.32
	12	0.13	(0.08)	0.10	(0.09)	0.10	(0.07)			0.22
	24	0.11	(0.09)	0.08	(0.05)	0.12	(0.06)			0.159
Alpha-linolenic acid C18:3, g *M (SD)*	0	1.7	(0.6)	1.7	(0.9)	2.0	(0.7)			0.23
	12	1.3_a_	(0.6)	1.4_b_	(0.8)	2.2_ab_	(1.0)			0.018
	24	1.3	(0.6)	1.5	(0.7)	2.3	(1.6)			0.160
EPA C20:5, g *M (SD)*	0	0.26	(0.33)	0.19	(0.22)	0.15	(0.20)			0.50
	12	0.25	(0.45)	0.18	(0.21)	0.22	(0.19)			0.50
	24	0.23	(0.25)	0.12	(0.14)	0.24	(0.21)			0.27
DPA C22:5, g *M (SD)*	0	0.09	(0.11)	0.05	(0.06)	0.09	(0.09)			0.52
	12	0.10	(0.12)	0.08	(0.09)	0.06	(0.07)			0.90
	24	0.08	(0.08)	0.05	(0.06)	0.08	(0.07)			0.48
DHA C22:6, g *M (SD)*	0	0.61	(0.72)	0.42	(0.47)	0.38	(0.46)			0.72
	12	0.70	(0.97)	0.50	(0.56)	0.53	(0.42)			0.69
	24	0.61	(0.55)	0.35	(0.39)	0.59	(0.48)			0.137
Cholesterol, mg *M (SD)*	0	316	(143)	314	(117)	292	(86)			0.91
	12	371	(198)	291	(159)	367	(137)			0.179
	24	334	(208)	265	(118)	338	(101)			0.20
Retinol equivalents, µg *M (SD)*	0	1180	(1826)	1151	(1333)	1036	(891)			0.69
	12	1074	(1131)	1022	(1117)	868	(525)			0.83
	24	618	(434)	770	(414)	864	(622)			0.178
Retinol, µg *M (SD)*	0	909	(1826)	854	(1319)	744	(936)			0.94
	12	695	(1051)	752	(1107)	612	(461)			0.83
	24	393	(284)	496	(419)	642	(661)			0.73
Beta carotene, µg *M (SD)*	0	2595	(3014)	2692	(1854)	2110	(1808)			0.39
	12	3709	(5093)	2532	(2433)	2413	(2554)			0.70
	24	2199	(2665)	2547	(1783)	1888	(2388)			0.122
Vitamin D, µg *M (SD)*	0	8	(6)	7	(4)	7	(4)			0.92
	12	8	(7)	7	(6)	7	(3)			0.34
	24	7	(4)	6	(4)	8	(4)			0.172
Vitamin E, mg *M (SD)*	0	11	(3)	10	(3)	12	(3)	1.23	0.04	0.30
	12	11	(6)	10	(5)	11	(3)			0.122
	24	11	(4)	9	(4)	11	(5)			0.34
Vitamin K, µg *M (SD)*	0	16	(8)	21	(17)	22	(17)			0.56
	12	22	(16)	17	(13)	27	(21)			0.165
	24	22	(16)	18	(12)	18	(11)			0.81
Thiamine, mg *M (SD)*	0	1.4	(0.4)	1.5	(0.6)	1.5	(0.4)	0.05	0.00	0.96
	12	1.3	(0.4)	1.4	(0.6)	1.3	(0.4)			0.92
	24	1.3	(0.4)	1.2	(0.5)	1.5	(0.7)			0.47
Riboflavin, mg *M (SD)*	0	1.6	(0.7)	1.6	(0.6)	1.6	(0.5)			0.93
	12	1.7	(0.8)	1.5	(0.9)	1.4	(0.3)			0.25
	24	1.4	(0.5)	1.3	(0.5)	1.4	(0.5)			0.79
Vitamin C, mg *M (SD)*	0	114	(66)	103	(47)	108	(47)			0.82
	12	121	(69)	120	(110)	114	(51)			0.70
	24	80	(50)	103	(61)	101	(73)			0.48
Niacin, mg *M (SD)*	0	21	(8)	19	(6)	23	(5)			0.27
	12	22	(9)	21	(9)	20	(6)	0.13	0.00	0.88
	24	18	(5)	18	(7)	22	(8)			0.184
Niacin equivalents, mg *M (SD)*	0	37	(11)	34	(10)	39	(7)			0.42
	12	38	(13)	35	(13)	35	(9)			0.85
	24	32	(7)	31	(11)	37	(11)	1.56	0.05	0.22
Vitamin B6, mg *M (SD)*	0	2.3	(0.7)	2.0	(0.6)	2.2	(0.6)	1.24	0.04	0.30
	12	2.3	(0.8)	2.1	(1.0)	2.0	(0.7)			0.51
	24	2.0	(0.6)	1.8	(0.7)	2.2	(0.7)			0.23
Vitamin B12, µg *M (SD)*	0	8.0	(10.9)	7.4	(9.0)	5.0	(2.1)			0.83
	12	7.7	(8.3)	6.2	(5.0)	6.2	(2.8)			0.63
	24	6.5	(5.5)	4.4	(2.2)	6.1	(2.1)			0.052
Phosphorous, mg *M (SD)*	0	1343	(339)	1297	(374)	1353	(274)	0.16	0.01	0.85
	12	1311	(378)	1251	(437)	1283	(281)	0.14	0.00	0.87
	24	1216	(274)	1142	(372)	1234	(280)	0.44	0.02	0.65
Folate, µg *M (SD)*	0	321	(232)	266	(81)	281	(108)			0.95
	12	321	(247)	284	(121)	293	(80)			0.61
	24	254	(66)	256	(112)	270	(80)			0.69
Iodine, µg *M (SD)*	0	127	(56)	103	(52)	124	(101)			0.26
	12	121	(46)	99	(58)	96	(62)			0.165
	24	122	(78)	93_a_	(51)	145_a_	(62)			0.031
Iron, mg *M (SD)*	0	11	(4)	11	(5)	11	(4)			0.98
	12	9	(3)	10	(4)	10	(2)	0.46	0.02	0.63
	24	9	(3)	9	(3)	15	(9)			0.21
Calcium, mg *M (SD)*	0	815	(336)	806	(282)	770	(206)	0.09	0.00	0.91
	12	789	(306)	691	(299)	707	(226)			0.42
	24	711	(241)	676	(324)	618	(185)			0.66
Potassium, mg *M (SD)*	0	3091	(740)	3077	(707)	2982	(691)	0.09	.00	0.91
	12	3200	(837)	2885	(930)	2876	(729)	0.88	0.03	0.42
	24	2830	(715)	2782	(863)	3039	(622)			0.43
Magnesium, mg *M (SD)*	0	314	(84)	314	(85)	318	(79)	0.01	0.00	0.99
	12	297	(87)	311	(115)	305	(65)	0.11	0.00	0.89
	24	293	(85)	288	(96)	296	(68)			0.85
Sodium, mg *M (SD)*	0	3226	(1020)	2836	(1209)	2762	(940)	0.98	0.03	0.38
	12	2620	(1054)	2746	(1297)	2807	(795)			0.68
	24	2606	(1194)	2459	(858)	2680	(793)			0.61
Selenium, mg *M (SD)*	0	50	(19)	49	(21)	46	(10)			0.93
	12	53	(22)	46	(23)	50	(14)			0.26
	24	51	(23)	41	(16)	51	(15)			0.068
Zinc, mg *M (SD)*	0	11	(3)	11	(4)	11	(2)	0.02	0.00	0.98
	12	10	(3)	10	(3)	10	(3)	0.04	0.00	0.96
	24	10	(2)	9	(4)	10	(3)			0.26
Fruit, g *M (SD)*	0	176	(150)	175	(115)	132	(133)			0.44
	12	207	(109)	171	(140)	198	(194)			0.37
	24	177	(143)	160	(112)	185	(182)	0.17	0.01	0.84
Vegetables legumes, g *M (SD)*	0	170	(113)	160	(103)	135	(124)			0.56
	12	233	(170)	160	(125)	159	(137)			0.34
	24	161	(111)	158	(100)	156	(130)			0.86
Potatoes, g *M (SD)*	0	111_a_	(123)	73	(53)	42_a_	(44)			0.049
	12	109_a_	(94)	68	(48)	39_a_	(33)			0.030
	24	68	(81)	72	(60)	84	(55)			0.43
Meat, g *M (SD)*	0	117	(99)	111	(73)	142	(84)			0.51
	12	131	(82)	98	(54)	118	(64)			0.35
	24	116	(71)	100	(71)	109	(79)			0.70
Meat products, g *M (SD)*	0	50	(64)	35	(50)	41	(48)			0.92
	12	26	(26)	36	(49)	18	(34)			0.44
	24	18	(43)	19	(33)	42	(52)			0.181
Fish shellfish, g *M (SD)*	0	46	(43)	56	(54)	34	(41)			0.44
	12	44	(59)	55	(51)	63	(54)			0.32
	24	43	(37)	40	(38)	68	(54)			0.19
Eggs, g *M (SD)*	0	24	(26)	29	(30)	27	(21)			0.75
	12	50	(49)	26_a_	(36)	57_a_	(34)			0.0086
	24	53	(60)	28	(29)	43	(32)			0.21
Soup sauce salad, g *M (SD)*	0	45	(78)	68	(72)	75	(154)			0.179
	12	41	(48)	86	(90)	80	(114)			0.179
	24	51	(59)	51	(71)	55	(58)			0.85
Water, g *M (SD)*	0	620	(574)	592	(524)	345	(365)			0.33
	12	358	(396)	499	(446)	334	(307)			0.45
	24	459	(387)	478	(417)	265	(236)			0.38
Diet nutrition preparations, g *M (SD)*	0	3	(11)	0	(0)	0	(0)			0.125
	12	2	(7)	2	(9)	0	(0)			0.74
	24	0	(0)	1	(7)	0	(0)			0.66
Additives baking ingredients, g *M (SD)*	0	14	(24)	12	(22)	12	(16)			0.94
	12	11	(15)	9	(17)	13	(23)			0.50
	24	26	(77)	12	(20)	11	(14)			0.46
Cereal grains, g *M (SD)*	0	249	(153)	185	(100)	243	(113)			0.22
	12	135	(126)	193	(104)	185	(89)			0.163
	24	199	(163)	162	(71)	152	(97)			0.63
Milk products, g *M (SD)*	0	241	(180)	258	(153)	211	(97)			0.67
	12	285	(202)	221	(164)	178	(133)			0.34
	24	240	(250)	226	(162)	175	(135)			0.64
Milk replacements, g *M (SD)*	0	0	(0)	0	(0)	0	(0)			0.99
	12	4	(17)	0	(0)	0	(0)			0.32
	24	8	(30)	0	(0)	0	(0)			0.25
Oil butter fat, g *M (SD)*	0	13	(11)	15	(11)	13	(11)			0.73
	12	5_ab_	(7)	12_a_	(9)	14_b_	(11)			0.0038
	24	9	(11)	15	(12)	16	(16)			0.080
Candy snacks ice cream soda jam, g *M (SD)*	0	61_a_	(44)	74	(66)	128_a_	(77)			0.047
	12	53	(71)	53	(64)	66	(42)			0.26
	24	47	(44)	53	(55)	60	(42)			0.63
Spirits, g *M (SD)*	0	7	(22)	3	(8)	8	(23)			0.92
	12	0	(0)	4	(18)	0	(0)			0.23
	24	1	(3)	1	(4)	0	(0)			0.67
Wine, g *M (SD)*	0	25	(53)	48	(74)	44	(64)			0.59
	12	45	(72)	48	(70)	57	(66)			0.80
	24	5	(19)	42	(73)	51	(84)			0.058
Beer, g *M (SD)*	0	22_a_	(54)	47	(97)	120_a_	(181)			0.013
	12	13_a_	(34)	21	(66)	79_a_	(90)			0.009
	24	0_ab_	(0)	37_a_	(62)	44_b_	(66)			0.026
Sweetened beverage, g *M (SD)*	0	143	(232)	69	(167)	62	(139)			0.142
	12	85	(178)	50	(138)	95	(221)			0.91
	24	45	(74)	39	(103)	43	(124)			0.63
Juice, g *M (SD)*	0	33	(56)	47	(70)	108	(106)			0.048
	12	41	(68)	35	(62)	56	(73)			0.49
	24	13	(35)	27	(59)	27	(60)			0.53
Water carbonated, g *M (SD)*	0	30	(71)	87	(174)	0	(0)			0.168
	12	27	(72)	73	(162)	16	(53)			0.43
	24	10	(39)	57	(143)	0	(0)			0.078
Coffee, g *M (SD)*	0	329	(361)	450	(269)	419	(314)			0.138
	12	320	(237)	446	(283)	402	(263)	1.32	0.04	0.28
	24	310	(186)	381	(264)	371	(293)			0.77
Tea, g *M (SD)*	0	135	(214)	125	(184)	69	(132)			0.55
	12	49	(116)	96	(142)	69	(88)			0.27
	24	43	(90)	69	(123)	83	(100)			0.40
Satiety before start of meal, RS *M (SD)*	0	−1.1	(0.5)	−1.1	(0.5)	−1.1	(0.5)	0.10	0.00	0.90
	12	−1.1	(0.6)	−1.0	(0.4)	−1.3	(0.5)	1.29	0.04	0.28
	24	−1.2	(0.5)	−1.0	(0.5)	−1.2	(0.4)	1.71	0.06	0.191
Satiety 30 min after start of meal, RS *M (SD)*	0	1.4	(0.5)	1.4	(0.5)	1.3	(0.5)	0.14	0.00	0.87
	12	1.4	(0.6)	1.4	(0.5)	1.3	(0.6)			0.55
	24	1.4	(0.6)	1.3	(0.5)	1.2	(0.4)			0.51
Satiety change during meal, RS *M (SD)*	0	2.5	(0.8)	2.4	(0.8)	2.4	(0.8)			0.81
	12	2.4	(1.0)	2.5	(0.8)	2.7	(0.9)			0.85
	24	2.7	(1.0)	2.3	(0.9)	2.4	(0.6)			0.55
Time between meals, HH:MM *M (SD)*	0	03:00	(00:59)	02:52	(00:39)	03:18	(00:42)	1.24	0.04	0.30
	12	03:16	(00:12)	02:54	(00:38)	03:18	(00:28)			0.26
	24	03:33	(00:59)	03:23	(00:47)	03:40	(00:51)	0.53	0.02	0.59
Satiety change to next meal, RS *M (SD)*	0	−0.9	(0.4)	−0.9	(0.6)	−0.8	(0.2)			0.64
	12	−0.8	(0.4)	−0.9	(0.4)	−0.8	(0.2)	0.30	0.01	0.74
	24	−0.9	(0.3)	−0.8	(0.3)	−0.7	(0.2)	0.83	0.03	0.44
Satiety quotient for energy, RS/MJ *M (SD)*	0	2.6	(1.8)	3.5	(4.7)	2.1	(1.6)			0.70
	12	2.8	(3.2)	3.6	(7.0)	1.8	(0.9)			0.66
	24	2.1	(1.0)	2.0	(1.4)	1.7	(0.7)			0.52
Satiety quotient for weight, RS/Kg *M (SD)*	0	6.6	(3.3)	6.5	(4.7)	6.6	(5.3)			0.75
	12	6.2	(4.5)	7.0	(4.8)	6.9	(5.1)			0.82
	24	7.1	(4.8)	6.6	(4.2)	6.4	(4.1)			0.96
Mean satiety change during meal/mean energy, RS/MJ *M (SD)*	0	2.6	(1.8)	3.5	(4.7)	2.1	(1.6)			0.70
	12	2.8	(3.2)	3.6	(7.0)	1.8	(0.9)			0.66
	24	2.1	(1.0)	2.0	(1.4)	1.7	(0.7)			0.52
Mean satiety change during meal/mean weight, RS/Kg *M (SD)*	0	6.5	(3.1)	6.4	(4.6)	6.5	(5.3)			0.73
	12	6.1	(4.5)	7.0	(4.8)	6.9	(5.1)			0.81
	24	7.1	(4.8)	6.5	(4.2)	6.3	(4.2)			0.94
Days, *n M (SD)*	0	3.9	(0.4)	4.0	(0.0)	4.0	(0.0)			0.36
	12	3.9	(0.2)	4.0	(0.0)	4.0	(0.0)			0.32
	24	4.0	(0.0)	4.0	(0.0)	4.0	(0.0)			0.99
Meals, *n M (SD)*	0	21	(7)	22	(5)	19	(3)			0.23
	12	19	(6)	21	(4)	18	(3)			0.090
	24	18	(4)	19	(3)	17	(4)	0.80	0.03	0.45
Meals per day, *n M (SD)*	0	5.4	(1.6)	5.6	(1.1)	4.8	(0.8)			0.24
	12	4.9	(1.5)	5.3	(1.1)	4.6	(0.7)			0.104
	24	4.5	(0.9)	4.6	(0.9)	4.3	(0.9)	0.80	0.03	0.45

*Note.* Means in a row sharing subscripts are significantly different from one another. EPA = eicosapentaenoic acid; DPA = docosapentaenoic acid; DHA = docosahexaenoic acid; RS = rating scale units.

## Discussion

There were no significant differences between participants based on group, exercise or diet allocation in primary ANOVA in change in the primary outcome waist circumference. The analyses were non-significant despite a more than 4 cm greater reduction in waist circumference at one year among participants allocated a diet without cereal grains and no exercise compared to participants allocated a diet with cereal grains and no exercise. This group difference was more than twice as large as was estimated in the pre-study power calculation, and its non-significance (*P* = 0.051) was most likely caused by too few participants and a greater than expected variability in the change in waist circumference. Participant recruitment was insufficient despite a prolonged study duration of over four years with intensive recruitment efforts via leaflets and advertisements. Variability, on the other hand, was unexpectedly high, with the standard deviation of the change in waist circumference ranging from 4 to 8 cm among the groups, compared to just under 3 cm in our previous study (Lindeberg *et al*., 2007). Nevertheless, the greater reduction in waist circumference among participants allocated a diet without cereal grains strengthens the possibility that dietary exclusion of cereal grains could be related to a greater reduction in waist circumference.

The ‘no grain’ dietary intervention encouraged exchanging cereal grain products for potatoes, root vegetables, fruit and other carbohydrate-rich foods. It was based on the findings indicating that Palaeolithic diets, which exclude cereal grains, reduce waist circumference more than control diets (mean difference = −2.5 cm, −2.5%) (de Menezes *et al*., 2019) and that beneficial effects on satiety regulation from cereal grain exclusion could be related to this larger reduction in waist circumference (Jönsson *et al*., 2015, 2010). While we are unaware of studies directly examining the effect of our specific dietary intervention, there is existing research on diets that restrict or decrease cereal grains, including high-fruit, Palaeolithic and those emphasising carbohydrate cellularity. For example, an RCT found no significant differences in visceral fat volume reductions over 6 and 12 months in individuals with obesity from a diet rich in cellular carbohydrates (like fruits, potatoes and vegetables) compared to a diet high in acellular carbohydrates (e.g., bread) (Sommersten *et al*., 2022). However, a 6-week RCT found larger weight loss (mean difference = −1.1 kg, −1.4%) in participants with overweight or obesity from a diet low in cereal grains and high in fruit compared to a high-cereal grain and low-fruit diet (Madero *et al*., 2011). Moreover, as mentioned above, multiple both short- and long-term RCTs on Palaeolithic diets found larger reductions in waist circumference (mean difference = −2.5 cm, −2.5%) (de Menezes *et al*., 2019), as well as other positive cardiometabolic outcomes such as improved insulin resistance, blood lipid profiles (total and low-density lipoprotein (LDL) cholesterol and triglycerides) and blood pressure compared to control diets (Sohouli *et al*., 2022). In previous studies, some downsides of Palaeolithic diets were lower calcium (Jönsson *et al*., 2009; Genoni *et al*., 2016; Titcomb *et al*., 2020) and iodine intake (Genoni *et al*., 2016; Manousou *et al*., 2018), primarily attributed to the exclusion of dairy foods and iodinated table salt – neither of which were restricted in our study. The ‘whole grain’ dietary intervention encouraged replacing refined cereal grain products with whole cereal grain products. The choice of comparing the ‘no grain’ with the ‘whole grain’ dietary intervention was based on our desire to compare two healthy diets that differed only in cereal grain content. Whole grain consumption, based on observational studies, is associated with higher micronutrient intake and improved diet quality (Mann *et al*., 2015; O’Neil *et al*., 2010; Smith *et al*., 2021). Moreover, short- and medium-term intervention studies with this ‘whole grain’ dietary intervention have shown beneficial cardiometabolic effects such as lowering total cholesterol, LDL cholesterol, HbA1c and C-reactive protein (Marshall *et al*., 2020), albeit with no changes in waist circumference or body fat percentage (Sadeghi *et al*., 2020).

The dietary interventions seem to have been successfully implemented, in so far as producing a significant difference between diets at one year in both cereal grain and whole grain intake, which, as planned, was achieved without an accompanying significant difference between diets in change in carbohydrate intake. Although larger reductions in mean cereal grain intake in the ‘no grain’ intervention groups would have been preferable, the −43% reduction at one year likely reflects a larger reduction earlier in the study. This would be consistent with the steady decline in subjective compliance ratings reported by the participants during the whole study. In fact, after two years, the cereal grain intake in the ‘no grain’ intervention groups had increased again, making the difference between diets non-significant. Over time, and despite monthly group sessions to encourage sustained dietary intervention adherence, this trend of decreasing intervention compliance was mirrored by a trend in all groups, including controls, for waist circumference to decrease initially, with the greatest reductions at 6 and 12 months, followed by a return towards baseline values at the end of the study at 24 months. Such a transitory pattern of effect from preventive interventions concurs with findings from previous studies (Ge *et al*., 2020).

Regarding the combination of diet and exercise, a recent meta-analysis on the effects of exercise intervention alone has shown that 15–60 min of structured aerobic exercise on 3–7 weekdays for 3–12 months reduces waist circumference by just over 3 cm (Armstrong *et al*., 2022). In comparison, the initial two-month long structured exercise intervention in this study with 120 min on two weekdays for one month followed by 120 min on one weekday for another month was a bit short, but a reduction of a couple of centimetres in waist circumference would not be an unreasonable expectation after three months. The physical activity prescription following the initial two-month structured exercise intervention has in a recent systematic review been shown to result in no significant intergroup differences in the change in the waist circumference (Onerup *et al*., 2019). The exercise intervention in this study could thus on its own be expected to produce an initial small difference in the waist circumference change of a couple of centimetres, followed by no further differences in the waist circumference change during the remainder of the study. The overall lack of significant differences in the changes in waist circumference in this study weakens the possibility of long-term effects from the combination of diet and exercise, which would be in line with results from short-term studies (Otten *et al*., 2017). Interpretive prudence is called for since the concurrent lack of significant differences in exercise intervention adherence could indicate an unsuccessful implementation of the exercise intervention. However, this possibility is less likely as the change in exercise intervention adherence at 12 months in the exercise intervention groups was a 27% increase in minutes per day spent at moderate-to-vigorous physical activity, corresponding to an increase of just over 6 min per day, which is higher than the increase of just over 2 min per day seen in a previous study on changes in objectively measured activity levels resulting from lifestyle interventions (Rockette-Wagner *et al*., 2022).

Interestingly, the control group had non-significantly larger decreases in waist circumference compared to several intervention groups at 6, 12 and 24 months. Although the aim of the study was to compare the dietary and exercise interventions with each other, the strong result in the control group should be carefully assessed as a source of further information. No instructions were given for the controls not to change their behaviour during the study, just that they would be monitored with measurements for two years. Therefore, it may well be that the controls sought other methods, by diet or exercise, to reduce their waist circumference. At 12 months, their change in exercise intervention adherence was a 16% increase in time spent at moderate-to-vigorous physical activity, putting them roughly midway between the exercise and no exercise intervention groups. At 24 months, their change in exercise intervention adherence was instead reversed into a 22% decrease, clearly below both the exercise and no exercise intervention groups. The physical activity changes in the controls were thus something of a mixed bag. Instead, their reduction in cereal grain intake was as large as among participants allocated to a diet without cereal grains and was also maintained for longer, that is, all the way to the end of the study. Thus, the controls in this study did not appear to be typical controls in the sense that they did not make any changes of their own regarding the study’s interventions. Instead, their strong results together with their decrease in cereal grain intake lends further strength to the possibility that dietary exclusion of cereal grains could be related to larger reductions in waist circumference.

## Limitations

The major limitation of this study comes from insufficient participant recruitment and underestimated variability in changes in the primary outcome waist circumference. Consequently, both the chances of detecting a true effect and the likelihood that a statistically significant result reflects a true effect are reduced. In hindsight, a multi-centre trial design would have facilitated sufficient participant recruitment. The underestimated variability of changes in waist circumference could, among other things, be related to the factorial design with its implementation of two different forms of interventions. Another limitation comes from the lack of monitoring of dietary and exercise intervention adherence early in the study, at three and six months. Such early monitoring would be preferable to get a better assessment of early intervention adherence and its effects on changes in waist circumference. In this study, monitoring at three and six months was refrained from in order not to discourage participation due to too many and cumbersome measurements. More frequent monitoring would also reduce the risk of participants improving their behaviour only when monitored. Monitoring during a study should therefore ideally be continuous, but that was not deemed feasible in this two-year-long study. More frequent monitoring might also help counter the accompanying potential of misreporting when using self-reported food intake methods such as the four-day weighed food records in this study (Ravelli and Schoeller, 2020). The low and declining reported total calorie intakes in this study possibly reflect such misreporting, which otherwise ought to have resulted in sustained decreases in waist circumference in all groups. Nevertheless, in our previous intervention studies with a Palaeolithic diet (Lindeberg *et al*., 2007; Jönsson *et al*., 2009), we observed even lower energy intakes accompanied by larger reductions in waist circumference, and this concordance supports the use of four-day diet records as valid measurements of dietary interventions despite its problems with misreporting.

A limitation in the generalisability of the study stems from its implementation in a comprehensive primary health care system that is available to all citizens. Replicating and implementing the study interventions in other settings with less comprehensive and accessible primary health care could require compensating strategies. These might involve promoting civil society and community involvement in health services (Lahariya *et al*., 2020) and securing sustained backing of policymakers to prioritise and fund health care centres with a broader range of services accessible to all citizens (Lahariya, 2020; Lahariya *et al*., 2020).

## Strengths

The longer duration and primary health care setting are strengths of this study. The longer duration improves our ability to study effect variation over time and enables the possibility of revealing an increased effect from combined diet and exercise compared to diet alone not seen in short-term interventions. The primary health care setting increases generalisability by studying these preventive measures in the part of the health care system with a preventive mission where these kinds of measures most likely would be implemented.

## Future research

Researchers should learn as much as possible from this study to avoid unethically repeating a study design that resulted in a larger than expected but non-significant result. Our results thus strengthen the possibility that dietary exclusion of cereal grains could be related to a greater reduction in waist circumference and weaken the possibility of long-term effects from diet and exercise combined. The dietary interventions seem to have been successfully implemented, but the implementation of the physical exercise intervention is more uncertain. The factorial trial design required more participants than could be recruited at one health station, and recruitment was possibly also made more difficult by the study being two years long. Therefore, a shorter one-year-long study should be performed, with the aim of only examining effects on waist circumference from a dietary intervention with and without cereal grains. Performing such a study in a primary health care setting still seems advantageous, for reasons mentioned before, but a multi-centre trial design should be implemented to ensure sufficient recruitment.

Other strategies to encourage study participation and completion should also be considered, such as community engagement, telemedicine and other rapidly evolving digital research technologies. The study protocol worked well regarding the dietary intervention delivery, data collection and monitoring of adverse events and could be used after being cleared of exercise intervention implementations and measurements. Among improvements to be considered would be more frequent and digitalised diet registrations. The funding required for the current study was relatively small since large parts were carried out within the framework of usual care at the health centre. A multi-centre trial, particularly if conducted in other settings without comprehensive public health care, might require additional resources.

## Conclusion

Group comparisons regarding the change in waist circumference were non-significant in the ANOVA despite a group difference more than double that estimated in the pre-study power calculation. The non-significance was probably caused by too few participants and a greater than expected variability in the change in waist circumference. The group difference strengthens the possibility that dietary exclusion of cereal grains could be related to a greater reduction in waist circumference. We provide recommendations for future research and suggest a follow-up one-year-long randomised controlled multi-centre primary health care intervention study only examining the effect of diet without cereal grains on reducing increased waist circumference.

## Data Availability

The dataset analysed during the current study is available from the corresponding author upon reasonable request.

## Declarations

The following declarations are listed here since they contain non-anonymous information.
